# Supplementary material for: Chromothripsis during telomere crisis is independent of NHEJ, and consistent with a replicative origin
Source: Genome Res. 2019 May;29(5):737–49. doi: 10.1101/gr.240705.118 (PMC6499312; doi:10.1101/gr.240705.118)
Supplement: Supplemental Material [file supp_gr.240705.118_Supplemental_file_1.zip › contigs/annotated_contigs/DB78/contig.2.DB78_length_413_mean_cov_11.4043583535.docx]

**DB78_length_413_mean_cov_11.4043583535**

```
CCCCACTGTGTCTGGAAGGTGGGCCATGGAACCAAAGAAGATTATTCTCAAGTCTTACGGTTTAATGTTGTTTGCCCTGTAGGTTTTCT
 >chr11:99230578-99230898 - E=2e-181
TTTTAATTGTCTATTTTTTAAATTTTTAATTTTTATGAGTATGTAGTGGGTATATGTATTTATGGGGTATATGAGATATTTTGACACAG

GCATACAAGGCATAATAATCACATCAGGGCAAATGAGGAATCCATCCCCTCATGTACTTTGATTTTTAATCCAAGTTGTTCATTCTGCC

TTTAAGTTTTTTTCATATATACTTGGTTTCAGTTACCCCTTTCTTCCTTCTGA|AGTAACAG|TCAGCTTGTGACTGTAATATTGCCCT
                                                              >chr11:99219870-99219955
TTCAGACACGATTGGGCACATTCAGGGTGGTCTGGCCATAGACTAATATTAGCTTTTCA- E=1e-36
```
